# Identification of two immunodominant and neutralizing linear B-cell epitopes exposed on the surface of the porcine deltacoronavirus spike protein

**DOI:** 10.1186/s13567-025-01690-x

**Published:** 2026-01-27

**Authors:** Xi Li, Huan Ye, Xinna Ge, Lei Zhou, Xin Guo, Jun Han, Yongning Zhang, Hanchun Yang

**Affiliations:** 1 State Key Laboratory of Veterinary Public Health and Safety, College of Veterinary Medicine, China Agricultural University, No. 2 Yuanmingyuan West Road, Haidian District, Beijing, 100193 China; 2 Key Laboratory of Animal Epidemiology of Ministry of Agriculture and Rural Affairs, College of Veterinary Medicine, China Agricultural University, Beijing, 100193 China

**Keywords:** Porcine deltacoronavirus (PDCoV), spike (S) protein, monoclonal antibodies (mAbs), neutralizing antibodies, B-cell epitopes

## Abstract

Porcine deltacoronavirus (PDCoV) is an enteropathogenic virus that causes severe diarrhea in pigs, particularly suckling piglets, and exhibits cross-species transmission with zoonotic potential. The S1 subunit of the viral spike (S) protein mediates cell entry and elicits neutralizing antibodies, making it an ideal target for diagnostics, prophylaxis, and therapeutics. Here, we produced two monoclonal antibodies (mAbs), B8F10 and G10C2, by immunizing BALB/c mice with a recombinant PDCoV S1 protein fused to a human IgG Fc fragment, expressed in an insect baculovirus system and purified using Protein A/G magnetic beads. Both mAbs can specifically recognize native PDCoV S protein in indirect immunofluorescence assays and western blot analyses under denaturing conditions, indicating their binding to linear epitopes. Isotyping classified both as IgG1/κ, and sequence analysis revealed distinct heavy-chain complementarity-determining regions (CDRs) but identical light-chain CDRs. Using truncated S1 proteins coupled with Pepscan ELISA, the B8F10 and G10C2 epitopes were precisely mapped to ^342^LETNFMCT^349^ and ^491^VINNTVVG^498^, respectively. These epitopes can be recognized by both mAbs and swine PDCoV antiserum, confirming their immunodominance. While global PDCoV strain alignments revealed high conservation of these epitopes, a V491A mutation within the G10C2-binding site abolished mAb binding. Structural analysis confirmed both epitopes are surface-exposed on S1. These findings demonstrate the diagnostic potential of these mAbs and the epitopes’ suitability as vaccine targets. Their high conservation suggests broad applicability, whereas the V491A mutation may represent an immune escape mechanism. These epitopes could serve as diagnostic markers, immunotherapeutics, or the foundation for epitope-based vaccines to induce protective immunity.

**Supplementary Information:**

The online version contains supplementary material available at 10.1186/s13567-025-01690-x.

## Introduction

Porcine deltacoronavirus (PDCoV) is an emerging enteropathogen primarily affecting pigs, with high pathogenicity in suckling piglets [1–4]. Clinically, infected piglets exhibit severe watery diarrhea, vomiting, dehydration, and fatal exhaustion [1–4], alongside histopathological lesions of atrophic enteritis, notably in the jejunum and ileum [3, 5]. Although PDCoV was initially identified in swine rectal swabs during a Hong Kong molecular epidemiological study [6], its pathogenicity was not confirmed until 2014, when it was associated with diarrheal outbreaks in USA piglets and sows [7]. Shortly after, the virus was successfully isolated from diarrheic pig intestinal contents using swine testicular (ST) and LLC porcine kidney (LLC-PK) cells [8]. Subsequent studies with diverse PDCoV isolates confirmed its causative role in swine diarrhea [2–4]. Since its initial USA outbreak [7], PDCoV has spread globally and now circulates in major pig-producing regions, including Canada, China, Korea, Thailand, Laos, Vietnam, Japan, Mexico, and Peru [9, 10]. It frequently causes mixed infections with other porcine enteric viruses, such as porcine epidemic diarrhea virus (PEDV), exacerbating acute gastroenteritis in neonatal piglets and inflicting substantial economic losses on the global swine industry [11, 12]. Despite its impact, effective therapeutic agents or vaccines against PDCoV remain limited [12]. Notably, PDCoV exhibits broad host tropism, infecting not only pigs but also chickens, turkeys, and calves under both natural and experimental conditions [13, 14]. In vitro studies further demonstrate its ability to efficiently replicate in cell lines from diverse species, including chickens, pigs, and humans [15, 16], underscoring its strong cross-species transmission potential [9, 16]. Of greater concern, PDCoV has recently been isolated from plasma samples of Haitian children with acute undifferentiated fever [17], signaling its zoonotic capacity and raising critical public health concerns.

Taxonomically, PDCoV is classified under the *Deltacoronavirus* genus within the *Coronaviridae* family (*Nidovirales* order) [18]. Its genome consists of a linear, nonsegmented, positive-sense single-stranded RNA (~25 kb), featuring 5′ and 3′ untranslated regions (UTRs), ORF1a and ORF1b (encoding polyproteins), four structural protein genes (spike [S], envelope [E], membrane [M], and nucleocapsid [N]), and two nonstructural genes (NS6, NS7) [11, 18]. Like other coronaviruses, the S protein of PDCoV plays a critical role in viral virulence [19], entry [15], tissue tropism [19], host range [15], and immune response [20]. This homotrimeric type I transmembrane protein consists of S1 and S2 subunits and is the primary structural protein on the virion surface [15, 21]. The S1 subunit contains two distinct domains—an N-terminal domain (S1-NTD) and a C-terminal domain (S1-CTD) [22, 23]—and exhibits a modular organization with four core domains (A–D), while the S2 subunit adopts an elongated helical structure with a C-terminal connector domain [20]. The S protein facilitates PDCoV entry by binding to its primary receptor, aminopeptidase N (APN), via the receptor-binding domain (RBD) in S1-CTD domain B [15, 23]. This interaction triggers S2-mediated fusion of the viral envelope with the host cell membrane, releasing the viral genome into the cytoplasm [20]. Notably, the PDCoV S protein exhibits high antigenicity and is abundantly expressed in both virions and infected cells [22], inducing robust humoral and cellular immune responses [24]. Importantly, all functional domains—including the RBD, S1-CTD, S1-NTD, and S2—have been demonstrated to elicit neutralizing antibodies [22, 24–26], establishing the S protein as a prime target for vaccine and therapeutic development.

Monoclonal antibodies (mAbs), particularly neutralizing mAbs, are powerful biological tools for diagnosing and treating infectious diseases [27, 28]. For instance, numerous mouse- or human-derived neutralizing mAbs targeting the severe acute respiratory syndrome coronavirus 2 (SARS-CoV-2) spike protein have proven crucial in coronavirus disease 2019 (COVID-19) treatment [27, 29–31]. Although the PDCoV S protein can induce neutralizing antibodies [22, 25, 26, 32], its immunodominant neutralizing epitopes remain largely uncharacterized. To date, only two linear B-cell epitopes (S280−288 and S506−513) have been identified in the S1-CTD region [26], with S280−288 serving as a neutralizing epitope recognized by mAb 4E-3, while S506−513 is a non-neutralizing epitope recognized by mAb 2A-12. Given the S protein’s strong antigenicity and role as the primary target for neutralizing antibodies, further identification of its neutralizing epitopes—particularly those in the RBD region—holds significant importance.

In this study, we immunized BALB/c mice with baculovirus-expressed recombinant PDCoV S1 protein and generated two neutralizing mAbs that specifically target PDCoV S1. We thoroughly characterized these mAbs by determining their isotypes, variable region sequences, target epitopes, spatial distribution, conservation, and immunogenicity.

## Materials and methods

### Virus and cells

The PDCoV strain CHN-HN-1601 (GenBank accession No. MG832584.1), originally isolated from the feces of a diarrheic piglet, has been maintained in our laboratory [33]. In addition, the following cell lines and bacterial strains were preserved in our laboratory: LLC-PK1 cells, *Spodoptera frugiperda* (Sf9) cells, murine myeloma SP2/0 cells, and *Escherichia coli* (*E. coli*) DH5α, BL21 (DE3), and DH10Bac competent cells. LLC-PK1 and SP2/0 cells were cultured at 37 °C in a humidified atmosphere containing 5% CO_2_, using Gibco Dulbecco’s modified eagle medium (DMEM) (Thermo Fisher Scientific, Carlsbad, CA, USA). The culture medium for LLC-PK1 cells was supplemented with 10% heat-inactivated fetal bovine serum (FBS) (Gibco, Carlsbad, CA, USA), while SP2/0 cells were maintained in DMEM supplemented with 20% fetal bovine serum (FBS). Sf9 cells were cultured at 28 °C in SIM SF expression medium (Sino Biological Inc., China).

### Construction of a recombinant bacmid expressing PDCoV S1-human IgG Fc fusion protein

The fusion gene, comprising the PDCoV S1 gene (nucleotides 1–1716 of the PDCoV CHN-HN-1601 strain) and the human IgG Fc fragment (nucleotides 776–1470 of *Homo sapiens* immunoglobulin heavy constant gamma 1; GenBank accession No. BC092518.1), linked by sequences encoding three flexible (GGGGS) peptides (Additional file 1), was synthesized by Sangon Biotech (Shanghai) Co., Ltd. The synthesized fusion gene was cloned into the pFastBac™ Dual baculovirus expression vector (Invitrogen, Carlsbad, CA) between the BamH I and EcoR I restriction sites via homologous recombination using the NEBuilder^®^ HiFi DNA Assembly Cloning Kit (New England BioLabs, Ipswich, MA). The resulting recombinant plasmid pFastBac-S1-Fc was sequenced for verification and then transformed into DH5α cells. A positive clone was subsequently transformed into DH10Bac cells for bacmid transposition. Positive recombinants were selected on Luria–Bertani agar plates containing 50 µg/mL kanamycin, 7 µg/mL gentamicin, 10 µg/mL tetracycline, 100 µg/mL Bluo-gal, and 40 µg/mL isopropyl-β-D-thiogalactoside (IPTG) via blue/white screening. Finally, the recombinant bacmid Bac-S1-Fc was extracted using the S.N.A.P. MidiPrep Kit (Invitrogen, Carlsbad, CA, USA), and its accuracy was verified by sequencing.

### Expression and purification of the recombinant S1-Fc fusion protein

The recombinant Bac-S1-Fc bacmid, encoding PDCoV S1 protein fused with human IgG Fc fragment, was transfected into Sf9 cells using CellFectin™ II Reagent (Thermo Fisher Scientific), followed by culture at 27 °C for 96 h. When cytopathic effects became evident (including growth arrest, budding vesicle formation, and eventual cell dissociation/lysis), the culture supernatant was centrifuged (500 × *g*, 5 min) to harvest first-passage (P1) recombinant baculovirus (Baculovirus-S1-Fc). This P1 stock was used to infect fresh Sf9 cells. Following 72 h cultivation at 27 °C, infected cells were pelleted by centrifugation. Cell pellets were lysed with NP-40 buffer and centrifuged (10 000 × *g*, 1 h, 4 °C). The S1-Fc fusion protein was then affinity-purified from the supernatant using Pierce™ Protein A/G Magnetic Beads (Thermo Fisher Scientific) according to the manufacturer’s instructions.

### Preparation of polyclonal and monoclonal antibodies against the PDCoV S1 protein

To prepare polyclonal antibodies against the PDCoV S1 protein, ten 6-week-old BALB/c mice were randomly divided into two groups (*n* = 5 per group). The experimental group received subcutaneous injections of purified S1-Fc protein (100 µg/mouse) emulsified with an equal volume of Freund’s adjuvant (Sigma-Aldrich, St. Louis, MO), while the control group was administered phosphate buffered saline (PBS) instead of S1-Fc protein. All mice were immunized via multi-site subcutaneous injection three times, with booster immunizations administered at weeks 2 and 4 after the primary immunization. In addition, mouse anti-S mAbs were generated as previously described [34–36], with minor modifications. Briefly, 6 week-old BALB/c mice were subcutaneously immunized in the dorsal region with 100 µg of purified S1-Fc protein emulsified 1:1 (v/v) with Freund’s complete adjuvant. Booster immunizations were administered twice at 2-week intervals using the same antigen emulsified with Freund’s incomplete adjuvant (Sigma-Aldrich). At 3 days after a final intraperitoneal boost with S1-Fc protein alone, mice were euthanized by cervical dislocation, and spleens were harvested. Splenocytes were fused with murine SP2/0 myeloma cells using polyethylene glycol 1450 (Sigma-Aldrich). Resulting hybridomas were selected in DMEM supplemented with 20% FBS, hypoxanthine-aminopterin-thymidine selection medium, and hypoxanthine-thymidine supplement. Hybridoma culture supernatants were subsequently screened for S protein-specific antibody secretion using indirect immunofluorescence assay (IFA). Positive clones were subcloned three times via limiting dilution. Stable hybridoma lines were then injected into female BALB/c mice for ascites production. The identification of isotype in prepared mAbs by mouse monoclonal antibody isotyping ELISA kit (Proteintech Group, Inc., Wuhan, China).

### B-cell epitope mapping of mAbs

To identify the B-cell epitopes recognized by each mAb, truncated forms of the PDCoV S1 gene were sequentially cloned into the pGEX-6p-1 vector between the BamH I and EcoR I/XhoI restriction sites, generating a series of overlapping truncations. Specifically, using pFastBac-S1-Fc as the template and the primer pairs listed in Additional file 2, five rounds of S1 truncation constructs were generated. The amplified fragments corresponding to each truncation were individually cloned into the pGEX-6p-1 vector to create recombinant plasmids. After confirming accuracy by sequencing, these plasmids were transformed into *E. coli* BL21 (DE3) and induced with 0.5 M IPTG at 37 °C for 6 h to express glutathione S-transferase (GST)-tagged truncated S1 fusion proteins. The truncated S1 proteins were then analyzed by western blot using rabbit anti-GST polyclonal antibody and the prepared mAbs as primary antibodies. Moreover, to validate the B-cell epitopes identified by truncation analysis, we synthesized five overlapping 8-mer peptides (with 6-amino acid overlaps between adjacent peptides) covering the initially mapped epitope region (Additional file 3). The peptides were synthesized by Sangon Biotech (Shanghai) Co., Ltd. The reactivity of these synthetic peptides with their respective mAbs was determined by Pepscan ELISA, as previously described [37]. Briefly, each of the synthetic peptides (5 μg/well) was coated overnight on a 96-well ELISA plate at 4 °C. The plates were then blocked for 2 h at 37 °C with 5% (w/v) skim milk in PBS, followed by three washes with PBST (PBS containing 0.05% Tween 20). Next, the plates were incubated with 100 μL of 1:100 diluted mAb or swine PDCoV antiserum at 37 °C for 1 h. After washing three times with PBST, bound antibodies were detected using horseradish peroxidase (HRP)-conjugated goat anti-mouse or anti-pig IgG (1:5000 dilution in PBST; Sigma-Aldrich). Following an additional 1 h incubation and three washes, the reaction was quantified by measuring absorbance at 450 nm (OD_450_) using 3,3’,5,5’-tetramethylbenzidine (Solarbio Life Sciences, Beijing, China) as the substrate. A sample was considered positive if its OD_450_ value was at least 2.1-fold higher than that of the negative control.

### IFA

Monolayers of Sf9 or LLC-PK1 cells were seeded in 6-well plates and inoculated with either the recombinant baculovirus expressing S1-Fc fusion protein or PDCoV strain CHN-HN-1601 at a multiplicity of infection (MOI) of 4 and 0.1, respectively. At 36 h postinoculation, the cells were washed twice with PBS and fixed with absolute ethanol at 4 °C for 20 min. After an additional PBS wash, the cells were blocked with 2% bovine serum albumin for 30 min at room temperature (RT), then incubated with primary antibodies at 37 °C for 1 h. The primary antibodies used included a mouse anti-human IgG Fc mAb (Biodragon Immunotechnologies, Suzhou, China), swine PDCoV antiserum, and the prepared mAbs. Following three PBS washes, the cells were incubated with secondary antibodies—fluorescein isothiocyanate (FITC)-conjugated goat anti-mouse IgG (1:200; ZSGB-BIO) or goat anti-pig IgG (1:200; Solarbio)—at 37 °C for 1 h. Finally, nuclei were stained with 4’,6-diamidino-2-phenylindole (DAPI), and fluorescence images were captured using a Nikon fluorescence microscope.

### Virus neutralization test (VNT)

A fluorescent focus VNT was performed to evaluate whether the PDCoV antiserum or prepared mAbs possess neutralizing activity and, if so, to determine their neutralization titers as previously described [38]. Briefly, all serum samples (PDCoV antiserum and negative control serum) were heat-inactivated at 56 °C for 30 min prior to use. Two-fold serial dilutions of PDCoV antiserum and mouse ascites-derived mAbs were prepared (1:40 to 1:1280). Negative control serum was generated by pooling sera from five naive mice followed by 1:2 dilution. For neutralization testing, 0.5 mL of viral inoculum was mixed with an equal volume of diluted serum or mAb. The regression experiment included four treatment groups: undiluted stock (200 TCID_50_/0.1 mL), 10^–1^, 10^–2^, and 10^–3^ dilutions. Control groups consisted of: (i) negative serum control (virus + negative serum) and (ii) blank control (DMEM only). All mixtures were incubated at 37 °C with 5% CO_2_ for 1 h, followed by DMEM supplementation in regression groups. Confluent LLC-PK1 monolayers in 96-well plates were washed thrice with PBS (0.01 M, pH 7.2). Quadruplicate wells were inoculated with 200 μL of each mixture. After 1 h adsorption (37 °C, 5% CO_2_), inoculum was removed and wells were washed. Maintenance medium (serum-free DMEM with 5 μg/mL trypsin) was added for 72 h incubation. Cells were fixed with ice-cold absolute ethanol (20 min, 4 °C), washed, and probed sequentially with: (i) in-house mouse anti-PDCoV N protein mAb 1A3 (1:2000) [33, 39], and (ii) FITC-conjugated goat anti-mouse IgG (Proteintech; 1:200). Following PBS washes (3 × 5 min), fluorescence was assessed by inverted microscopy. The neutralization titer was defined as the maximal serum dilution achieving > 90% reduction in fluorescent foci (Reed-Muench method). All samples were tested in triplicate, with data presented as mean titer ± SD. Neutralizing antibody levels were determined for both serum and mAb groups following establishment of regression curves with controls.

### Western blot

Total cellular proteins extracted from Sf9 cells infected with recombinant baculovirus expressing S1-Fc fusion protein or *E. coli* BL21(DE3) expressing PDCoV S1 (or its truncation mutants) were separated by 12% sodium dodecyl sulfate–polyacrylamide gel electrophoresis (SDS-PAGE) and transferred to polyvinylidene fluoride (PVDF) membranes. After blocking with 5% skim milk (2 h, RT), membranes were incubated with prepared mAbs or PDCoV antiserum (1 h, RT), followed by five PBST washes. Membranes were then probed with HRP-conjugated goat anti-mouse IgG or anti-pig IgG (1 h, RT). After five additional PBST washes, target proteins were visualized using enhanced chemiluminescence reagent (Vigorous Biotechnology, Beijing, China) and imaged with a Bio-Rad Gel Imager System (Hercules, CA).

### Determination of mAb heavy- and light-chain variable regions

The variable regions of each mAb’s heavy and light chains were PCR-amplified using our previously described primer sets [35]. Amplicons containing the variable regions were cloned into pEASY-Blunt Zero vector (TransGen Biotech, Beijing, China). The positive recombinant plasmids were sequenced by Tsingke Biotechnology Co., Ltd. (Beijing, China) using M13F/R universal primers. The resulting sequences were then analyzed with the NovoPro Online Tool [40] to identify the complementarity-determining regions (CDRs)—CDR-H1, CDR-H2, CDR-H3 for the heavy chain and CDR-L1, CDR-L2, CDR-L3 for the light chain—as well as the intervening framework regions (FRs), based on the Kabat numbering scheme.

### Spatial localization of the identified epitopes

The three-dimensional structure of PDCoV S1 protein was determined through homology modeling using the Swiss-Model platform. Model01 was selected for subsequent analysis on the basis of optimal quality assessment parameters, including a 99.10% sequence identity with its template structure—the 3.5 Å-resolution cryo-electron microscopy (cryo-EM) structure of PDCoV S glycoprotein trimer (PDB: 6BFU) [20]. Structural characterization was performed using PyMOL (v4.6.0, Schrödinger, LLC), with particular focus on two identified epitopes. Domain architecture was annotated as follows: N-terminal domain (NTD, residues 52−277) and C-terminal domain (CTD, residues 302−422) according to Shang et al. [21], with the receptor-binding domain (RBD, residues 300−419) localized within the CTD region as previously reported [23].

### Statistical analysis

Statistical analyses were performed using GraphPad Prism software (Version 8.0; La Jolla, CA, USA). Data are presented as the mean ± SD from three independent experiments. Statistical significance was defined as follows: *P < 0.05, **P < 0.01, ***P < 0.001, and ****P < 0.0001.

## Results

### Expression and purification of the recombinant S1-Fc fusion protein

**Figure 1:**
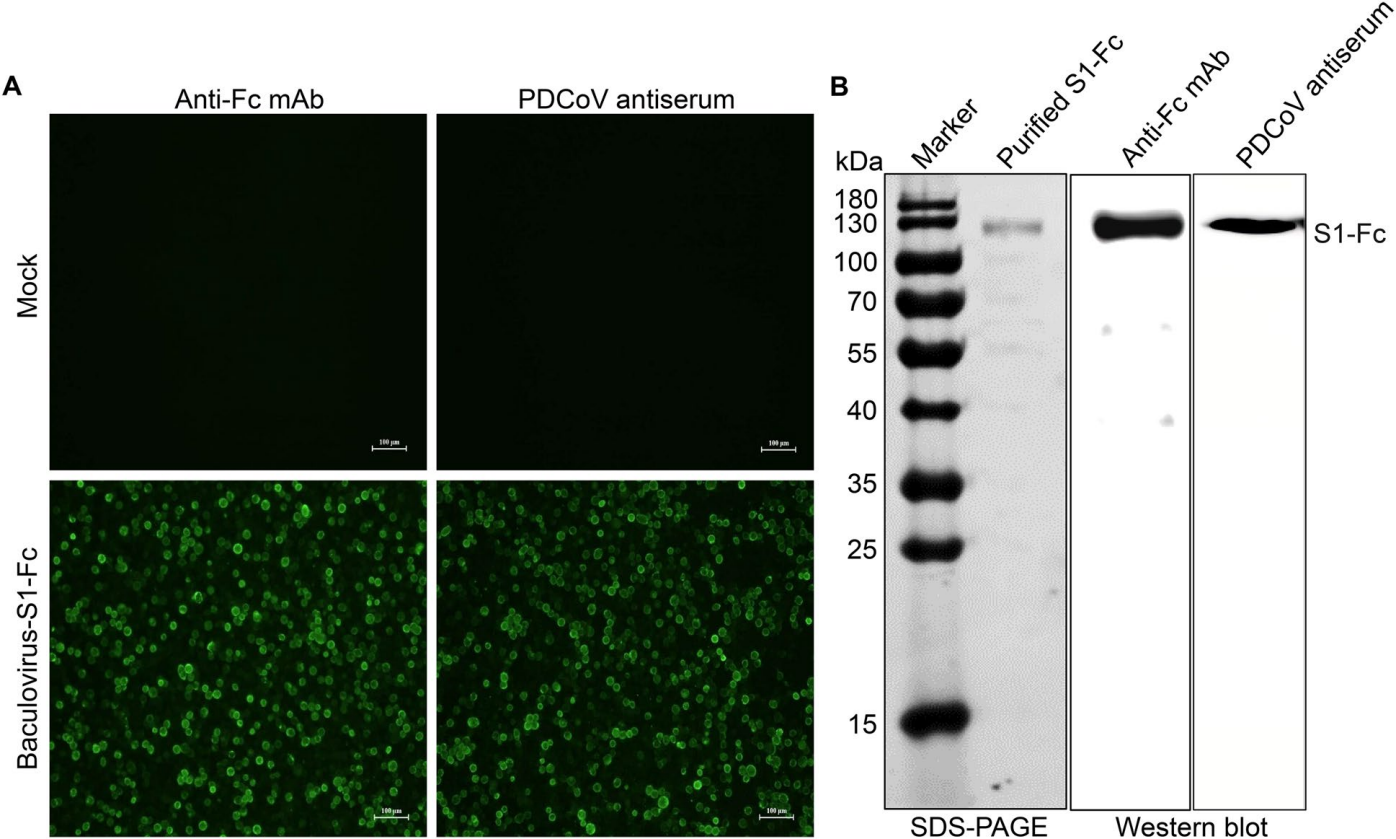
**Identification of the PDCoV-S1-Fc fusion protein expressed by the insect baculovirus expression system**. **A** Detection of PDCoV S1-Fc fusion protein in recombinant baculovirus-infected Sf9 cells by IFA. Sf9 cells were either mock-infected or infected with recombinant baculovirus (Baculovirus-S1-Fc) at an MOI of 4 for 48 h. After fixation, the cells were probed with either an Fc-specific mAb or swine PDCoV antiserum, followed by immunostaining with FITC-conjugated goat anti-mouse IgG or goat anti-pig IgG secondary antibodies, and visualized by fluorescence microscopy. **B** SDS-PAGE and western blot analysis of the S1-Fc protein purified using Protein A/G magnetic beads.

To confirm successful expression of the S1-Fc fusion protein in Sf9 cells, we first performed an IFA to assess reactivity of Baculovirus-S1-Fc-infected cells with Fc-specific mAb and swine PDCoV antiserum. Specific cytoplasmic staining was observed in infected cells, while control cells showed no signal (Figure 1A). The S1-Fc protein was then affinity-purified using Protein A/G magnetic beads via its Fc tag. Sodium dodecyl sulfate–polyacrylamide gel electrophoresis (SDS-PAGE) analysis showed a prominent ~120 kDa band matching the predicted molecular weight (Figure 1B), which was further confirmed by western blot to react with both anti-Fc mAb and PDCoV antiserum (Figure 1B). These results demonstrate the successful S1-Fc expression and purification.

### Evaluation of neutralizing antibody induction by the PDCoV S1 protein in vivo

**Figure 2:**
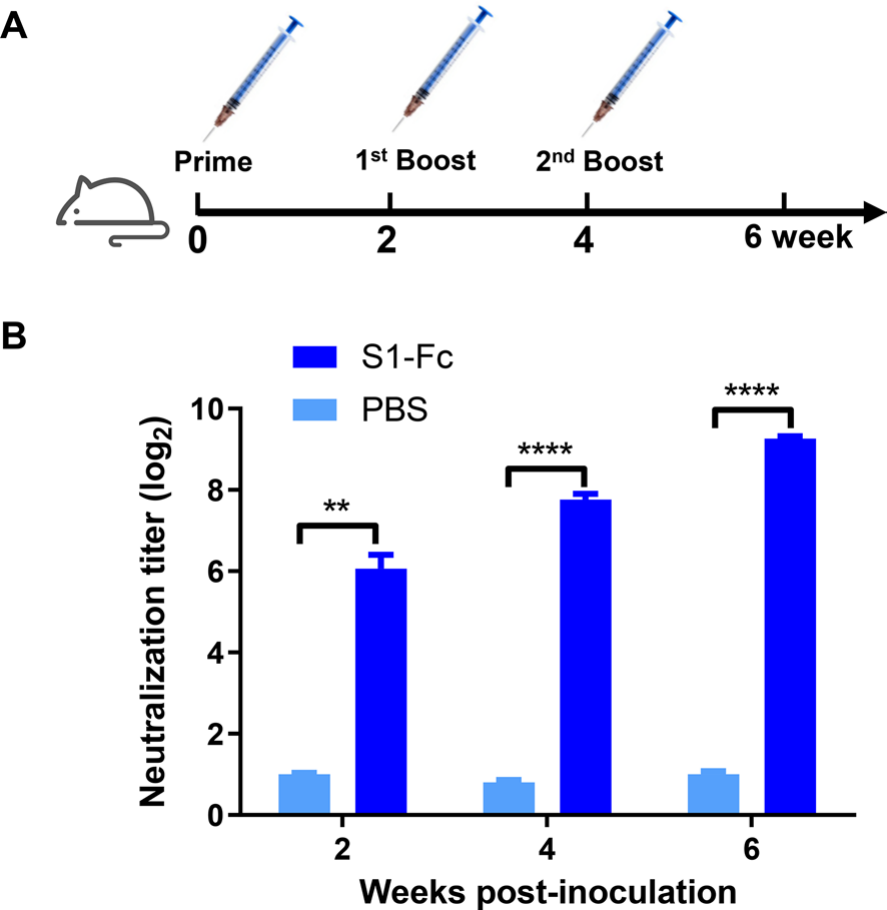
**Evaluation of the PDCoV S1 protein’s potential to induce neutralizing antibody production in vivo**. **A** Immunization timeline for BALB/c mice. BALB/c mice received subcutaneous immunization with 100 µg of purified S1-Fc fusion protein emulsified in Freund’s adjuvant, while controls received PBS. Booster immunizations were given at weeks 2 and 4 postprimary immunization. **B** Determination of PDCoV-neutralizing antibody titers in the antisera of mice immunized with the S1-Fc fusion protein. Mouse sera were collected at 2, 4, and 6 weeks postinitial immunization and analyzed for PDCoV-neutralizing activity using a fluorescent focus virus neutralization test. Data are shown as mean ± SD of three independent experiments (two-way analysis of variance (ANOVA); *p < 0.05, **p < 0.01, ***p < 0.001, ****p < 0.0001).

To assess the neutralizing antibody-inducing potential of PDCoV S1 protein in vivo, BALB/c mice were immunized subcutaneously three times with the purified S1-Fc fusion protein according to the schedule shown in Figure 2A. Mouse sera were collected at 2, 4, and 6 weeks postpriming and analyzed for PDCoV-neutralizing activity using a fluorescent focus VNT assay. As shown in Figure 2B, S1-Fc-immunized mice, but not PBS controls, developed PDCoV-neutralizing serum. Specifically, high levels of neutralizing antibodies were detected as early as 2 weeks after prime immunization. The neutralizing antibody titers progressively increased following booster immunizations, reaching 1:67 ± 18, 1:217 ± 11, and 1:614 ± 26 at weeks 2, 4, and 6 postprimary immunization, respectively. These findings indicate the PDCoV S1 protein possesses the ability to elicit neutralizing antibody responses in the host.

### Generation and characterization of mAbs targeting the PDCoV S1 protein

**Figure 3:**
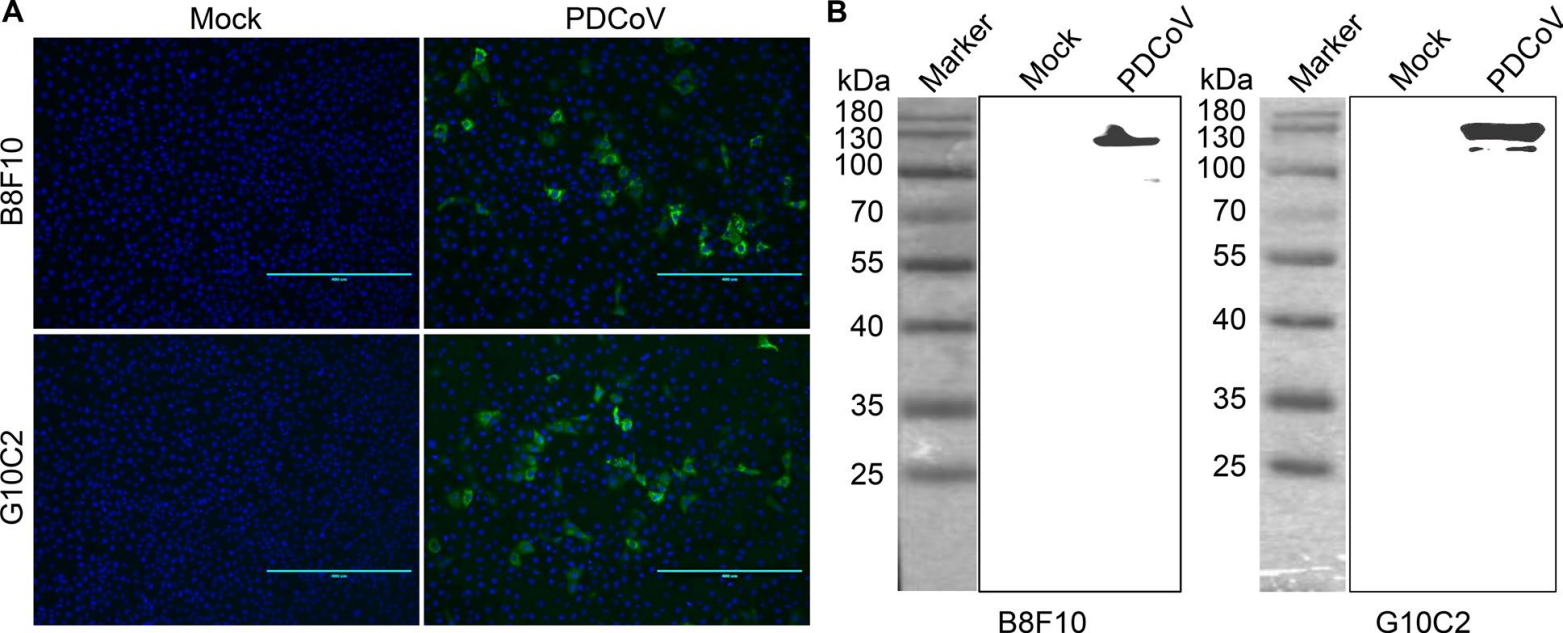
**Evaluation of S1 mAb immunoreactivity with PDCoV under infection conditions**. **A** IFA analysis of S1 mAbs immunoreactivity with PDCoV. LLC-PK1 cells were mock-infected or infected with PDCoV strain CHN-HN-1601 (MOI = 0.1) for 36 h, then fixed and probed with mAbs B8F10 or G10C2. After immunostaining with FITC-conjugated goat anti-mouse IgG and counterstaining nuclei with DAPI, fluorescence microscopy was performed. **B** Western blot analysis of S1 mAbs immunoreactivity with PDCoV. LLC-PK1 cells, mock-infected or infected with PDCoV CHN-HN-1601 (MOI = 0.1, 36 h), were lysed and analyzed by western blot using mAbs B8F10 and G10C2 as primary antibodies and HRP-conjugated goat anti-mouse IgG as the secondary antibody.

Through IFA screening followed by three rounds of subcloning, we successfully obtained two monoclonal hybridoma cell clones (B8F10 and G10C2) stably secreting anti-PDCoV S1 mAbs. These clones were expanded in vivo via intraperitoneal injection into BALB/c mice to generate antibody-rich ascites. Both mAbs demonstrated robust immunoreactivity, exhibiting specific recognition of PDCoV in both IFA and western blot analyses. In IFA, the mAbs produced a distinct cytoplasmic staining pattern exclusively in PDCoV-infected cells, with no signal observed in mock-infected cells (Figure 3A). Western blot analysis under denaturing conditions revealed a ~130 kDa protein band exclusively in PDCoV-infected cell lysates (Figure 3B), consistent with the theoretical molecular weight of the native S protein under denaturing conditions. The detection of this band under denaturation suggests that the epitopes recognized by B8F10 and G10C2 are linear. Further characterization confirmed that both mAbs belong to the IgG1/κ isotype.

### Neutralization activity analysis of S1 mAbs and determination of their neutralizing titers

**Figure 4:**
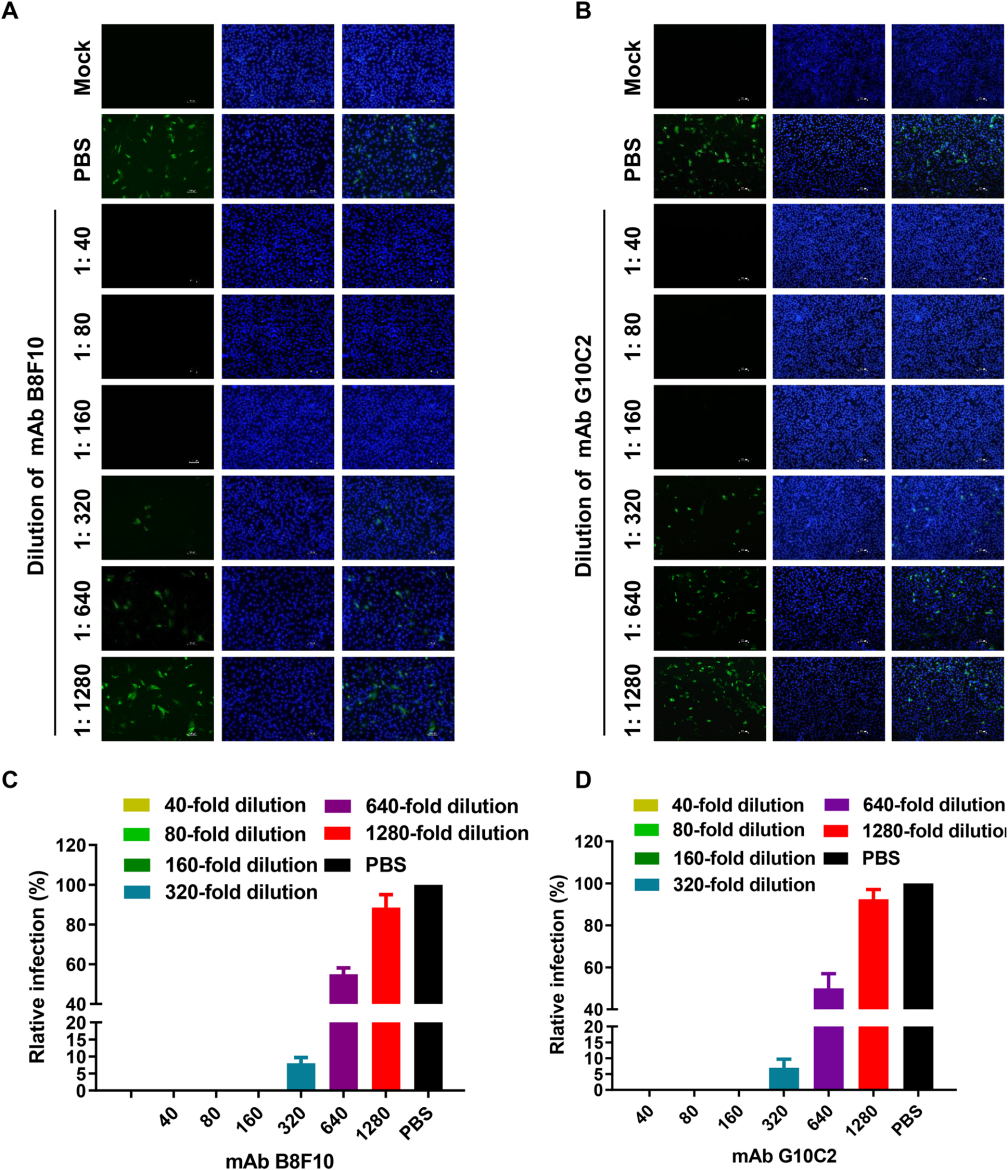
**Analysis of neutralization activity and determination of neutralizing titers for S1 mAbs**. **A**, **B** Representative results of the fluorescent focus neutralization test demonstrating the neutralizing activity of mAbs B8F10 and G10C2. **C**, **D** Neutralizing titers of mAbs B8F10 and G10C2 as measured by fluorescent focus neutralization test.

The neutralizing activity of the two prepared mAbs was evaluated using a fluorescent focus VNT, measuring their ability to inhibit PDCoV infection in LLC-PK1 cells. As shown in Figures 4A and B, mAbs B8F10 and G10C2 exhibited comparable neutralizing activity against PDCoV infection. Quantitative analysis based on the relative proportion of infected LLC-PK1 cells revealed end point neutralizing titers of 1:398 ± 34 for B8F10 and 1:453 ± 25 for G10C2, respectively (Figures 4C and D).

### Mapping B-cell epitopes of the PDCoV S1 protein recognized by the prepared mAbs

**Figure 5:**
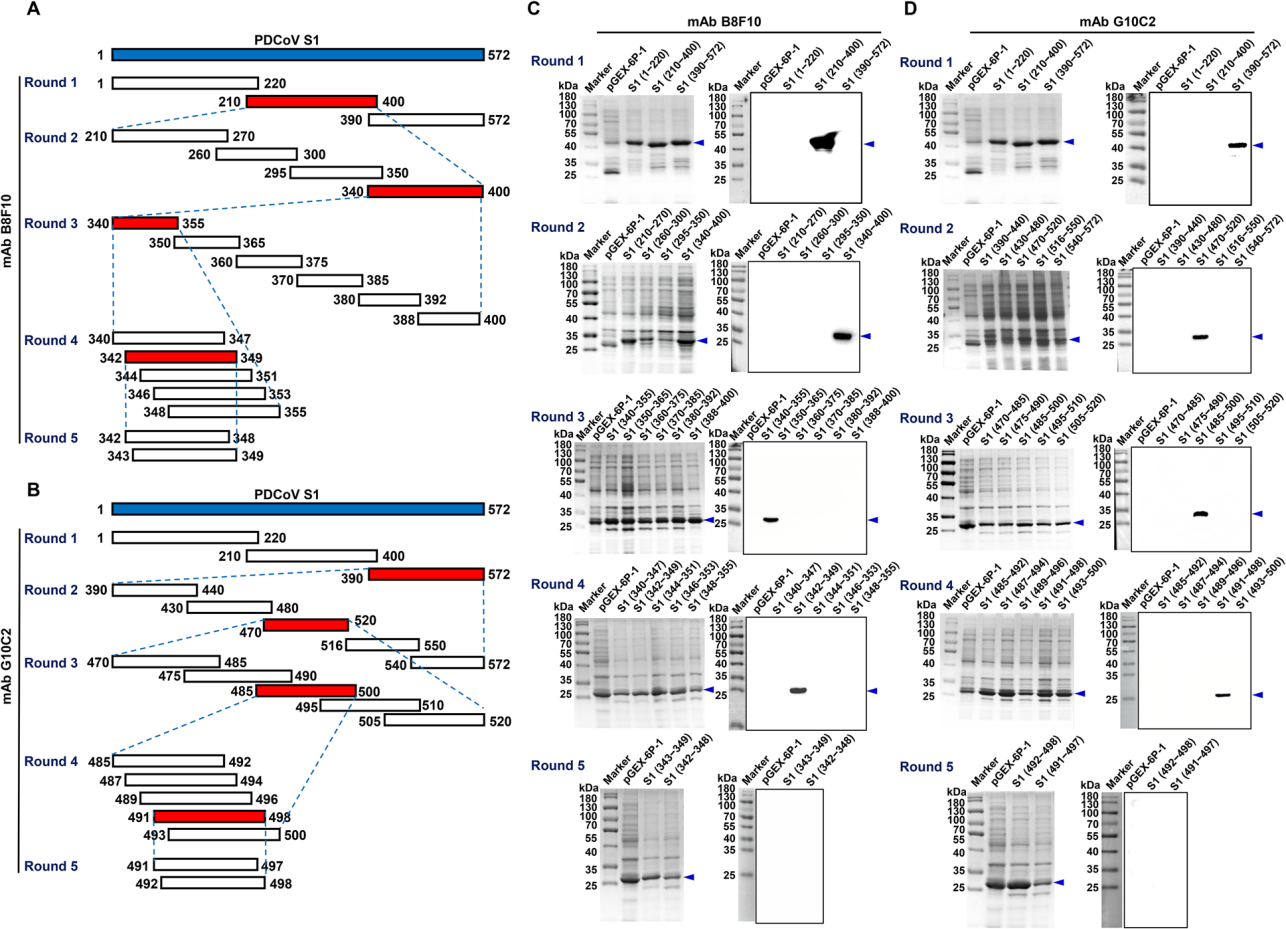
**Identification of B-cell epitopes recognized by the two prepared mAbs against the PDCoV S1 protein**. **A**, **B** Strategy for constructing five rounds of truncated forms of GST-tagged PDCoV S1 protein. Numbers indicate the amino acid positions in the full-length PDCoV S1 protein. The target protein is marked with a blue arrow. The red box highlights the PDCoV S1 protein region recognized by the mAb. **C**, **D** Western blot analysis of the reactivity of mAbs B8F10 and G10C2 with each truncated GST-tagged PDCoV S1 protein.

The epitopes recognized by the two mAbs were mapped using five rounds of progressive truncation of the GST-tagged PDCoV S1 protein (Figure 5A), followed by western blot analysis (Figure 5B). In the first round, mAbs B8F10 and G10C2 recognized the 210−400 and 390−572 amino acid (aa) fragments, respectively. In the second round, they recognized the 340−400 and 470−520 aa fragments, respectively. In the third round, recognition was narrowed to the 340−355 and 485−500 aa fragments. In the fourth round, the epitopes were further localized to the 342−349 and 491−498 aa fragments. In the fifth round, neither mAb recognized the further truncated constructs (343–349, 342−348, 492−498, and 491−497) (Figure 5C). These results localize the B8F10 and G10C2 epitopes to residues 342−349 and 491−498, respectively.

### Validation of the identified B-cell epitopes of PDCoV S1 protein by ELISA

**Figure 6:**
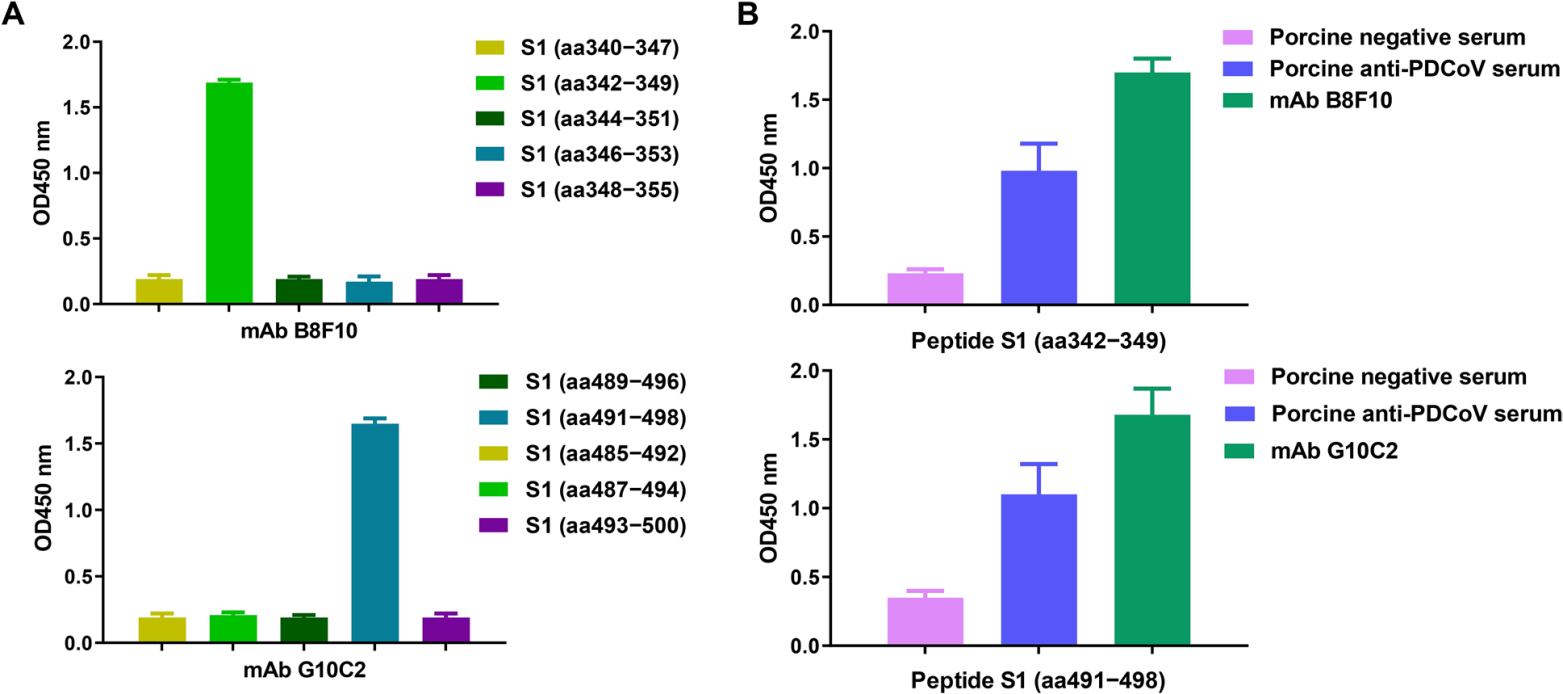
**Immunoreactivity profiling of mAb-targeted epitopes by ELISA. A** Pepscan ELISA evaluation of B8F10 and G10C2 binding to five overlapping octapeptides. **B** ELISA quantification of synthetic epitope peptide binding to mAbs (B8F10 and G10C2) or porcine antiserum.

To validate the truncation-identified B-cell epitopes, we synthesized five overlapping octapeptides (with a 6-aa overlap) spanning each candidate region for both mAbs. Pepscan ELISA revealed specific reactivity only between peptide 342−349 and mAb B8F10, and between peptide 491−498 and mAb G10C2 (Figure 6A), confirming that residues ^342^LETNFMCT^349^ and ^491^VINNTVVG^498^ represent the respective B-cell epitopes recognized by these mAbs. Notably, both epitope peptides exhibited reactivity not only with their corresponding mAbs (B8F10 or G10C2) but also with swine PDCoV antiserum (Figure 6B). These findings demonstrate that the two identified epitopes are immunodominant regions of the PDCoV S1 protein capable of eliciting neutralizing antibody responses during PDCoV infection in pigs

### Conservation analysis of the identified epitopes

**Figure 7:**
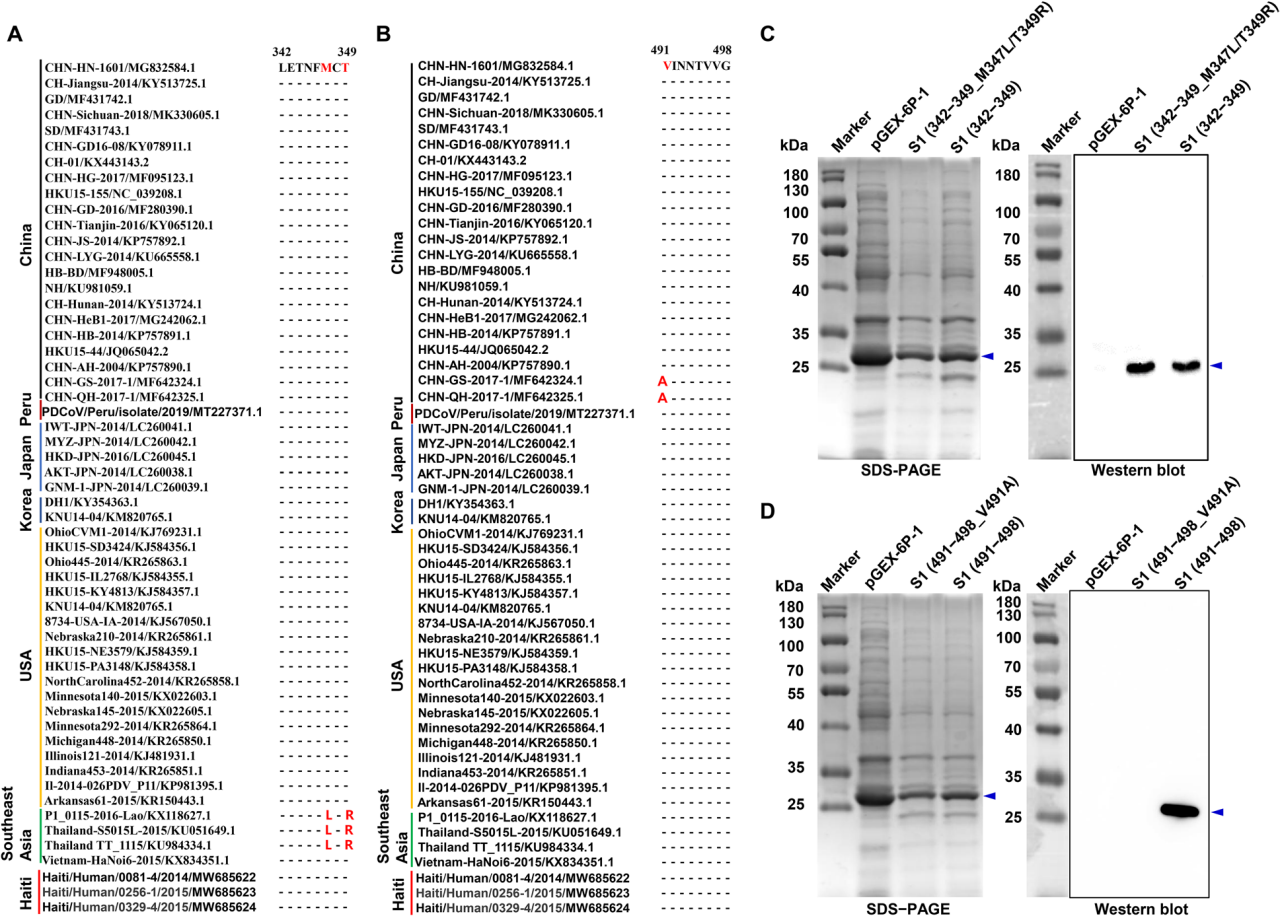
**Cross-strain conservation analysis of the two identified B-cell epitopes on the PDCoV S1 protein**. **A** Conservative analysis of the epitope ^342^LETNFMCT^349^ recognized by mAb B8F10 among different ASFV strains. **B** Conservative analysis of the epitope ^491^VINNTVVG^498^ recognized by mAb G10C2 among different ASFV strains. **C** Analysis of the impact of double mutations (M347L/T349R) within the epitope ^342^LETNFMCT^349^ on the reactivity of mAb B8F10 with PDCoV S1. The wild-type and mutant epitopes were fused to GST, solubly expressed in *E. coli* BL21(DE3), and purified from the cell lysates via glutathione agarose affinity chromatography. **D** Analysis of the impact of the single mutation (V491A) within the epitope ^491^VINNTVVG^498^ on the reactivity of mAb G10C2 with PDCoV S1. The wild-type and mutant epitopes were fused to GST, solubly expressed in *E. coli* BL21(DE3), and purified from the cell lysates via glutathione agarose affinity chromatography. The target protein is marked with a blue arrow.

Comparative analysis of S1 genes from 56 representative PDCoV strains across nine countries in the GenBank database revealed that both mAb-targeted epitopes were relatively conserved (Figures 7A and B). The B8F10-targeted epitope (^342^LETNFMCT^349^) displayed only two mutations (M347L and T349R) in two Thai strains (KU051649.1 and KU984334.1) and one Laotian strain (KX118627.1). In contrast, the G10C2-targeted epitope (^491^VINNTVVG^498^) was more highly conserved, with only a single V491A substitution in two Chinese strains (MF642324.1 and MF642325.1). To assess whether these mutations affected mAb binding, we constructed prokaryotic expression plasmids carrying the M347L/T349R (B8F10 epitope) or V491A (G10C2 epitope) mutations. Following expression in *E. coli* BL21(DE3) and induction with IPTG, western blot analysis revealed that the M347L/T349R double mutations did not disrupt B8F10 binding (Figure 7C). Strikingly, however, the V491A single mutation completely abolished G10C2 reactivity (Figure 7D), demonstrating that residue 491 is essential for antigenic recognition by this mAb.

### Variable region sequencing of mAb heavy and light chains

The heavy- and light-chain variable regions of each mAb were PCR-amplified and sequenced. Nucleotide sequences of mAbs B8F10 and G10C2 were deposited in National Center for Biotechnology Information (accession numbers OR494597–OR494598 and OR494595–OR494596, respectively; Table 1). The corresponding amino acid sequences of the variable heavy (V_H_) and light (V_L_) chains were analyzed using the Kabat numbering scheme to delineate their CDR and FR regions. The V_H_ and V_L_ domains exhibit a conserved modular architecture, structured as FR1-CDR1-FR2-CDR2-FR3-CDR3-FR4. For visual distinction, FRs are depicted in black, while CDRs are color-coded (CDR1: red, CDR2: blue, CDR3: green). As summarized in Table 1, the two mAbs have distinct heavy-chain CDR sequences but identical light-chain CDR sequences. This information is crucial for the direct expression of engineered antibodies that specifically recognize the PDCoV S protein.

**Table 1:**
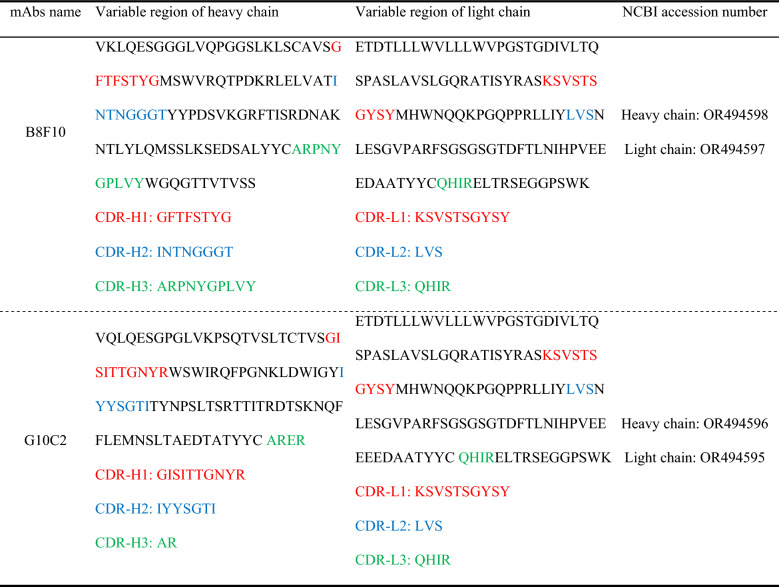
**Amino acid sequences of the heavy- and light-chain variable regions of the two mouse anti-PDCoV S1 mAbs**

FRs: black; CDR1: red; CDR2: blue; CDR3: green.

### Spatial distribution of the two identified epitopes

**Figure 8:**
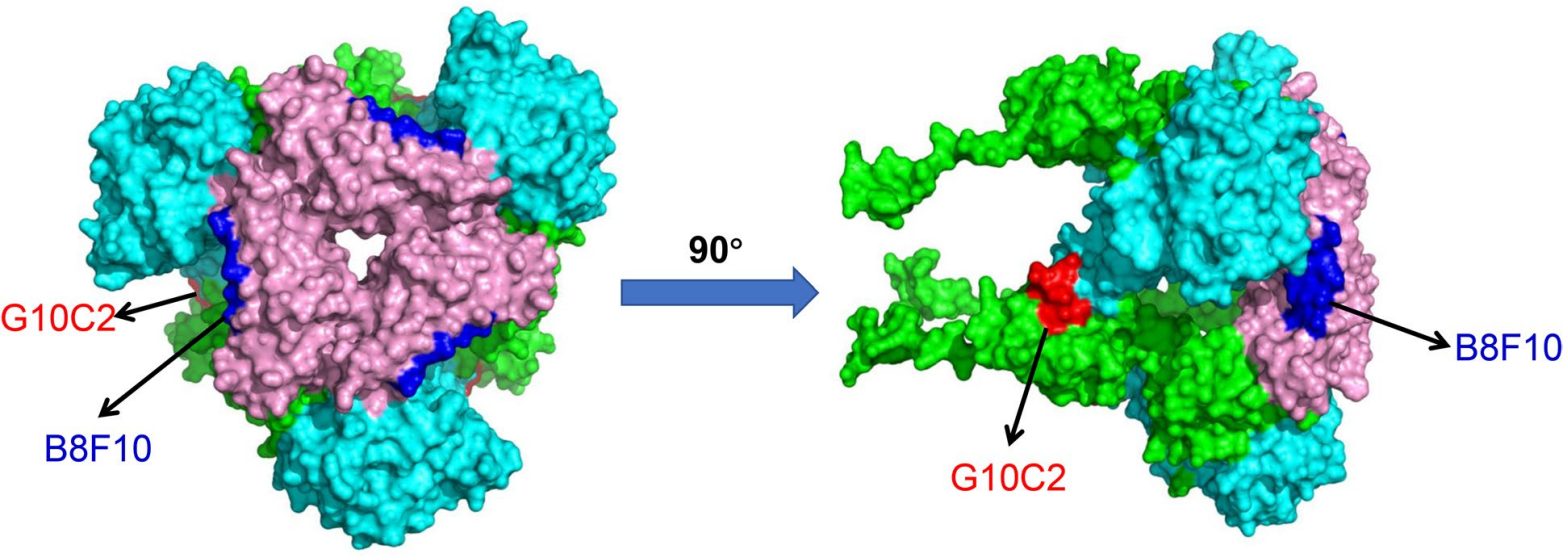
**The spatial distribution of the two identified neutralizing epitopes on the PDCoV S trimer.** Color-coded structural representation: the N-terminal domain (NTD, residues 52−277) is shown in cyan, while the C-terminal domain (CTD, residues 302−422) appears in pink. Monoclonal antibody B8F10 binding sites are highlighted in blue, and G10C2 epitopes are marked in red. All remaining amino acid residues are displayed in green.

To further elucidate the spatial localization of the identified epitopes ^342^LETNFMCT^349^ and ^491^VINNTVVG^498^ on the PDCoV S protein, we conducted homology modeling (Swiss-Model) using the cryo-EM-resolved PDCoV S protein structure as reference [20], followed by structural visualization analysis (PyMOL). Our results demonstrate that both epitopes recognized by mAbs B8F10 and G10C2 are fully exposed on the PDCoV S protein surface, indicating high antibody-binding accessibility (Figure 8).

## Discussion

PDCoV has emerged as a significant threat to global swine husbandry, causing severe diarrhea in suckling piglets and incurring substantial economic losses [41]. Its demonstrated ability to cross species barriers—including potential zoonotic transmission, as evidenced by recent isolation from Haitian children—further underscores the urgency for targeted interventions [13, 14]. The S protein of coronaviruses, particularly its S1 subunit, plays a crucial role in host cell entry and is the primary inducer of neutralizing antibodies, making it a prime target for immunotherapy, vaccine development, and diagnostic design [42–44]. A prime example is SARS-CoV-2, for which multiple neutralizing mAbs targeting the S protein—generated via murine hybridoma technology or isolated from human B cells—have demonstrated potent antiviral efficacy in vitro and in vivo. Several of these mAbs have received emergency use authorization or full regulatory approval, facilitating their clinical deployment for targeted COVID-19 interventions [27, 29–31]. For PDCoV, domain B of the S1-CTD functions as the RBD, which interacts with APN to facilitate viral entry into host cells [15]. Previous studies have highlighted the immunogenicity of the S1 subunit, with neutralizing antibodies targeting regions critical for receptor engagement [22, 25, 26]. However, before our study, only a few linear B-cell epitopes in PDCoV S1—such as the neutralizing epitope S280–288 and the non-neutralizing epitope S506–513—had been identified [26]. In addition, a conserved conformational epitope recognized by the PDCoV-neutralizing mAb 4A6 was recently mapped to the S1 protein, though its precise location remains unknown [45]. In the present study, we identified two novel neutralizing epitopes, ^342^LETNFMCT^349^ and ^491^VINNTVVG^498^, on the surface of the PDCoV S1 subunit. These epitopes are immunodominant, capable of eliciting neutralizing antibodies, and highly conserved across strains. Their discovery not only expands the repertoire of functionally critical regions in the PDCoV S protein but also offers promising targets for therapeutic, diagnostic, and vaccine development. Notably, the epitopes identified in our study differ from the two previously reported PDCoV S1 epitopes, S280–288 (^280^FYSDPKSAV^288^) and S506–513 (^506^TENNRFTT^513^), both of which reside in the S1-CTD but map to a distinct region [26]. This diversity of neutralizing epitopes within S1 suggests that the PDCoV S protein harbors multiple immunodominant regions that may synergistically contribute to protective immunity. The presence of these distinct epitopes could be leveraged to develop multivalent vaccines, potentially broadening immune response coverage and enhancing potency. Furthermore, the immunodominance of the two identified epitopes is supported by their reactivity not only with their corresponding mAbs (B8F10 and G10C2) but also with swine PDCoV antiserum in our ELISA assays (Figure 6). This indicates that these linear epitopes on the PDCoV S1 protein are natural targets of the humoral immune response during infection in pigs and are capable of eliciting neutralizing antibodies. Based on these properties—high specificity, affinity, conservation, and confirmed immunodominance—the mAbs we generated are excellent candidates for developing diagnostic immunoassays. Specifically, they hold significant promise for use in rapid antigen test kits for point-of-care diagnosis, which are vital tools for timely outbreak control and disease surveillance.

A critical criterion for selecting vaccine and diagnostic targets lies in their sequence conservation across viral strains [46, 47]. Our analysis of 56 global PDCoV strains revealed high conservation of both ^342^LETNFMCT^349^ and ^491^VINNTVVG^498^ epitopes, with only rare mutations observed. The B8F10 epitope exhibited two mutations (M347L and T349R) in a few Southeast Asian strains without compromising antibody binding, whereas the G10C2 epitope showed a single V491A mutation only in two Chinese strains that completely abolished antibody binding (Figure 7). This mutation suggests a potential antibody escape mechanism, underscoring the need for enhanced surveillance of such variants to ensure the long-term efficacy of epitope-based intervention strategies. The high conservation of these epitopes across geographically diverse strains implies they may be under strong functional selective pressure, possibly owing to their critical roles in maintaining spike protein structure or mediating receptor binding. Given the global prevalence of PDCoV, this conservation makes them ideal universal targets for pan-PDCoV vaccines and diagnostic reagents, holding significant application value.

Virus neutralization tests demonstrated that both B8F10 and G10C2 exhibited potent neutralizing activity against PDCoV, with neutralizing titers of 1:398 ± 34 and 1:453 ± 25, respectively (Figures 4C and D). Mechanistically, neutralization likely occurs by blocking S1-APN interactions, as both epitopes are surface-exposed on the S1 subunit. B8F10 targets a linear epitope (^342^LETNFMCT^34^⁹) within the RBD core, which directly binds porcine APN [15], while G10C2 recognizes an epitope (^491^VINNTVVG^498^) in subdomain SD’’—a region potentially involved in spike stability or cooperative receptor binding [20, 21]. These findings are consistent with structural studies of other coronaviruses (e.g., SARS-CoV-2, Middle East respiratory syndrome coronavirus), where RBD-directed neutralizing antibodies sterically hinder receptor binding or destabilize the prefusion S1 conformation [48, 49]. Further structural analyses of antibody-epitope complexes could elucidate the precise neutralization mechanisms. Notably, both epitopes were recognized by swine PDCoV antiserum, confirming their immunodominance during natural infection. Their strong immunogenicity highlights their potential as vaccine targets for eliciting protective neutralizing antibodies against PDCoV. For vaccine development, epitope-based vaccines offer advantages over full-length protein vaccines, including reduced risk of antibody-dependent enhancement and focused induction of neutralizing responses [46, 47]. The conserved, neutralizing nature of these epitopes makes them ideal candidates for such vaccines. Preliminary studies with other coronaviruses have shown that multiepitope vaccines, incorporating multiple neutralizing regions, can induce robust protection [50, 51]. Combining the epitopes identified here with previously reported ones (e.g., S280–288) could further enhance vaccine efficacy. In addition, the identified epitopes hold significant promise for PDCoV diagnostics. Linear epitopes are particularly valuable for serological assays (e.g., ELISA) owing to their stability under denaturing conditions, ensuring consistent detection across sample types [52]. Given their conservation, serological assays targeting ^342^LETNFMCT^349^ or ^491^VINNTVVG^498^ could enable broad-spectrum detection of PDCoV exposure, aiding in epidemiological surveillance.

While our present study provides valuable insights, several limitations should be addressed. First, the neutralizing activity of the mAbs was evaluated in vitro; in vivo studies—such as passive transfer experiments in piglets—are needed to confirm protection against PDCoV-induced disease. Second, the structural basis of antibody–epitope interactions, including how they block APN binding, remains to be elucidated via cryo-EM or X-ray crystallography. Furthermore, molecular dynamics (MD) simulations could be employed in future work to probe the conformational dynamics and binding stability of these antibody-epitope complexes. Third, the potential for these epitopes to induce cellular immunity, which is critical for long-term protection, requires investigation. Future work should focus on developing epitope-based vaccines incorporating ^342^LETNFMCT^349^ and ^491^VINNTVVG^498^, alone or in combination with other identified neutralizing epitopes, and testing their efficacy in piglets. Fourth, during the electroblotting step, wherein separated proteins are transferred from the gel to a polyvinylidene fluoride (PVDF) membrane, the transfer buffer lacks a reducing agent but contains methanol. Methanol promotes the dissociation of SDS from the protein complexes. Consequently, some proteins may undergo partial refolding, potentially restoring conformational epitopes that could then be recognized by antibodies [53, 54]. To eliminate interference from such conformational epitopes and conclusively demonstrate that the target epitopes for our mAbs (B8F10 and G10C2) are linear, we synthesized short peptides corresponding to the immunoreactive protein regions identified by immunoblotting. A positive signal in the ensuing peptide ELISA directly confirms that both mAbs recognize linear epitopes (Figure 6). In addition, monitoring the emergence of escape mutants—particularly those with V491A or other mutations in these epitopes—will be essential for maintaining vaccine effectiveness. Finally, exploring the role of these epitopes in cross-species transmission could inform strategies to prevent zoonotic spillover.

In summary, the discovery of two conserved, neutralizing, and immunodominant linear B-cell epitopes on the PDCoV S1 subunit advances our understanding of PDCoV immunology and offers valuable targets for diagnostics, immunotherapy, and vaccine development. Their surface exposure, high conservation, and capacity to elicit neutralizing antibodies highlight their potential for designing interventions to combat PDCoV in swine and reduce its zoonotic risk. Future studies should validate their in vivo protective efficacy and characterize their structural interactions with host receptors to translate these findings into practical applications.

## Supplementary Information

**Additional file 1 Construction details of the pFastBac™ Dual baculovirus expression plasmid for the PDCoV S1-Fc fusion protein.**

**Additional file 2 Primers used for the construction of recombinant plasmids.**

**Additional file 3 Information on the two sets of overlapping 8-mer peptides (6-amino-acid stagger) synthesized for epitope mapping.**

## Data Availability

Data will be made available on reasonable request.
