# Supplementary material for: Identification of two immunodominant and neutralizing linear B-cell epitopes exposed on the surface of the porcine deltacoronavirus spike protein
Source: Vet Res. 2026 Jan 27;57:33. doi: 10.1186/s13567-025-01690-x (PMC12918246; doi:10.1186/s13567-025-01690-x)
Supplement: Supplementary file 1 — Additional file 1 Construction details of the pFastBac™ Dual baculovirus expression plasmid for the PDCoV S1-Fc fusion protein. [file 13567_2025_1690_MOESM1_ESM.docx]

**
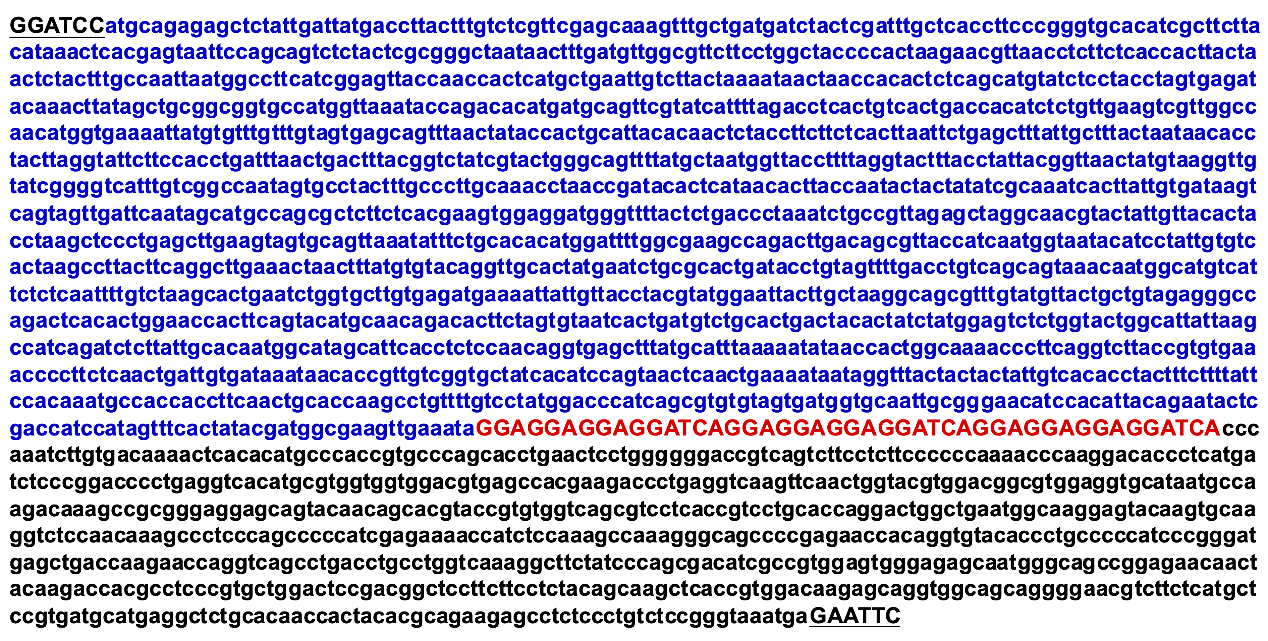
**

**Additional file 1. Construction details of the pFastBac™ Dual baculovirus expression plasmid for the PDCoV S1-Fc fusion protein.** The underlined upstream and downstream sequences denote the *BamH* I and *EcoR* I restriction sites, respectively. Blue indicates the S1 gene (nt 1‒1716) of PDCoV strain CHN-HN-1601 (GenBank accession no: MG832584.1). Black indicates the human IgG Fc fragment (nt 776‒1470; GenBank accession no: BC092518.1). Red indicates the nucleotide sequence encoding three flexible linker peptides (GGGGS).
