# Supplementary material for: Identification of two immunodominant and neutralizing linear B-cell epitopes exposed on the surface of the porcine deltacoronavirus spike protein
Source: Vet Res. 2026 Jan 27;57:33. doi: 10.1186/s13567-025-01690-x (PMC12918246; doi:10.1186/s13567-025-01690-x)
Supplement: Supplementary file 2 — Additional file 2 Primers used for the construction of recombinant plasmids. [file 13567_2025_1690_MOESM2_ESM.docx]

**Additional file 2.** Primers used for the construction of recombinant plasmids.

| Primer name | Primer sequences (5ʼ→3ʼ) * | Restriction enzymes |
| --- | --- | --- |
| S1 (1–572) F | TTCCAGGGGCCCCTGGGATCCATGCAGAGAGCTCTATTGATTATG | *BamH* Ⅰ |
| S1 (1–572) R | CTCGAGTCGACCCGGGAATTCTATTTCAACTTCGCCATCGTATA | *EcoR* I |
| S1 (1–220) F | TTCCAGGGGCCCCTGGGATCCATGCAGAGAGCTCTATTGATTATG | *BamH* Ⅰ |
| S1 (1–220) R | CTCGAGTCGACCCGGGAATTCGTTAACCGTAATAGGTAAAG | *EcoR* I |
| S1 (210–400) F | TTCCAGGGGCCCCTGGGATCCTACCTTTTAGGTACTTTACC | *BamH* Ⅰ |
| S1 (210–400) R | CTCGAGTCGACCCGGGAATTCCCTTAGCAAGTAATTCCATAC | *EcoR* I |
| S1 (390–572) F | TTCCAGGGGCCCCTGGGATCCATTGTTACCTACGTATGGAATTAC | *BamH* Ⅰ |
| S1 (390–572) R | CTCGAGTCGACCCGGGAATTCTATTTCAACTTCGCCATCGTATA | *EcoR* I |
| S1 (210–270) F | TTCCAGGGGCCCCTGGGATCCTACCTTTTAGGTACTTTACC | *BamH* Ⅰ |
| S1 (210–270) R | CTCGAGTCGACCCGGGAATTCCTGGCATGCTATTGAATCAACTAC | *EcoR* I |
| S1 (260–300) F | TTCCAGGGGCCCCTGGGATCCGATAAGTCAGTAGTTGATTC | *BamH* Ⅰ |
| S1 (260–300) R | CTCGAGTCGACCCGGGAATTCCTTAGGTAGTGTAACAATAG | *EcoR* I |
| S1 (295–350) F | TTCCAGGGGCCCCTGGGATCCATTGTTACACTACCTAAGC | *BamH* Ⅰ |
| S1 (295–350) R | CTCGAGTCGACCCGGGAATTCACCTGTACACATAAAGTTAG | *EcoR* I |
| S1 (340–400) F | TTCCAGGGGCCCCTGGGATCCTTCAGGCTTGAAACTAACT | *BamH* Ⅰ |
| S1 (340–400) R | CTCGAGTCGACCCGGGAATTCCCTTAGCAAGTAATTCCATAC | *EcoR* I |
| S1 (340–355) F | TTCCAGGGGCCCCTGGGATCCTTCAGGCTTGAAACTAACT | *BamH* Ⅰ |
| S1 (340–355) R | CTCGAGTCGACCCGGGAATTCCAGATTCATAGTGCAACCTG | *EcoR* I |
| S1 (350–365) F | TTCCAGGGGCCCCTGGGATCCGGTTGCACTATGAATCTGCG | *BamH* Ⅰ |
| S1 (350–365) R | CTCGAGTCGACCCGGGAATTCTGACAGGTCAAAACTACAGG | *EcoR* I |
| S1 (360–375) F | TTCCAGGGGCCCCTGGGATCCTGTAGTTTTGACCTGTCAG | *BamH* Ⅰ |
| S1 (360–375) R | CTCGAGTCGACCCGGGAATTCTTGAGAGAATGACATGCC | *EcoR* I |
| S1 (370–385) F | TTCCAGGGGCCCCTGGGATCCGGCATGTCATTCTCTCAATTTTG | *BamH* Ⅰ |
| S1 (370–385) R | CTCGAGTCGACCCGGGAATTCACAAGCACCAGATTCAGTGC | *EcoR* I |
| S1 (380–392) F | TTCCAGGGGCCCCTGGGATCCACTGAATCTGGTGCTTGTG | *BamH* Ⅰ |
| S1 (380–392) R | CTCGAGTCGACCCGGGAATTCGGTAACAATAATTTTCATCTC | *EcoR* I |
| S1 (388–400) F | TTCCAGGGGCCCCTGGGATCCAAAATTATTGTTACCTACG | *BamH* Ⅰ |
| S1 (388–400) R | CTCGAGTCGACCCGGGAATTCCCTTAGCAAGTAATTCCATAC | *EcoR* I |
| S1 (390–440) F | TTCCAGGGGCCCCTGGGATCCATTGTTACCTACGTATGG | *BamH* Ⅰ |
| S1 (390–440) R | CTCGAGTCGACCCGGGAATTCGACTCCATAGATAGTGTAG | *EcoR* I |
| S1 (430–480) F | TTCCAGGGGCCCCTGGGATCCGATGTCTGCACTGACTACAC | *BamH* Ⅰ |
| S1 (430–480) R | CTCGAGTCGACCCGGGAATTCGACCTGAAGGGTTTTGCCAG | *EcoR* I |
| S1 (470–520) F | TTCCAGGGGCCCCTGGGATCCAAAAATATAACCACTGGCAAAACC | *BamH* Ⅰ |
| S1 (470–520) R | CTCGAGTCGACCCGGGAATTCGAAAGTAGGTGTGACAATAG | *EcoR* I |
| S1 (516–550) F | TTCCAGGGGCCCCTGGGATCCGTCACACCTACTTTCTTTTATTC | *BamH* Ⅰ |
| S1 (516–550) R | CTCGAGTCGACCCGGGAATTCCGCAATTGCACCATCACTAC | *EcoR* I |
| S1 (540–572) F | TTCCAGGGGCCCCTGGGATCCCCCATCAGCGTGTGTAGTG | *BamH* Ⅰ |
| S1 (540–572) R | CTCGAGTCGACCCGGGAATTCTATTTCAACTTCGCCATCG | *EcoR* I |
| S1 (470–485) F | TTCCAGGGGCCCCTGGGATCCAAAAATATAACCACTGGCAAAACC | *BamH* Ⅰ |
| S1 (470–485) R | CTCGAGTCGACCCGGGAATTCGGTTTCACACGGTAAGACC | *EcoR* I |
| S1 (475–490) F | TTCCAGGGGCCCCTGGGATCCGGCAAAACCCTTCAGGTCTTA | *BamH* Ⅰ |
| S1 (475–490) R | CTCGAGTCGACCCGGGAATTCAATCAGTTGAGAAGGGGTTTC | *EcoR* I |
| S1 (485–500) F | TTCCAGGGGCCCCTGGGATCCACCCCTTCTCAACTGATTG | *BamH* Ⅰ |
| S1 (485–500) R | CTCGAGTCGACCCGGGAATTCGATAGCACCGACAACGGTGT | *EcoR* I |
| S1 (495–510) F | TTCCAGGGGCCCCTGGGATCCACCGTTGTCGGTGCTATCAC | *BamH* Ⅰ |
| S1 (495–510) R | CTCGAGTCGACCCGGGAATTCCCTATTATTTTCAGTTGAG | *EcoR* I |
| S1 (505–520) F | TTCCAGGGGCCCCTGGGATCCTCAACTGAAAATAATAGGTTTAC | *BamH* Ⅰ |
| S1 (505–520) R | CTCGAGTCGACCCGGGAATTCGAAAGTAGGTGTGACAATAG | *EcoR* I |
| S1 (340–347) F | ***GATCC*** TTCAGGCTTGAAACTAACTTTATG ***C*** | *BamH* Ⅰ |
| S1 (340–347) R | ***TCGAG*** CATAAAGTTAGTTTCAAGCCTGAA ***G*** | *Xho* I |
| S1 (342–349) F | ***GATCC*** CTTGAAACTAACTTTATGTGTACA ***C*** | *BamH* Ⅰ |
| S1 (342–349) R | ***TCGAG*** TGTACACATAAAGTTAGTTTCAAG ***G*** | *Xho* I |
| S1 (344–351) F | ***GATCC*** ACTAACTTTATGTGTACAGGTTGC ***C*** | *BamH* Ⅰ |
| S1 (344–351) R | ***TCGAG*** GCAACCTGTACACATAAAGTTAGT ***G*** | *Xho* I |
| S1 (346–353) F | ***GATCC*** TTTATGTGTACAGGTTGCACTATG ***C*** | *BamH* Ⅰ |
| S1 (346–353) R | ***TCGAG*** CATAGTGCAACCTGTACACATAAA ***G*** | *Xho* I |
| S1 (348–355) F | ***GATCC*** TGTACAGGTTGCACTATGAATCTG ***C*** | *BamH* Ⅰ |
| S1 (348–355) R | ***TCGAG*** CAGATTCATAGTGCAACCTGTACA ***G*** | *Xho* I |
| S1 (485–492) F | ***GATCC*** ACCCCTTCTCAACTGATTGTGATA ***C*** | *BamH* Ⅰ |
| S1 (485–492) R | ***TCGAG*** TATCACAATCAGTTGAGAAGGGGT ***G*** | *Xho* I |
| S1 (487–494) F | ***GATCC*** TCTCAACTGATTGTGATAAATAAC ***C*** | *BamH* Ⅰ |
| S1 (487–494) R | ***TCGAG*** GTTATTTATCACAATCAGTTGAGA ***G*** | *Xho* I |
| S1 (489–496) F | ***GATCC*** CTGATTGTGATAAATAACACCGTT ***C*** | *BamH* Ⅰ |
| S1 (489–496) R | ***TCGAG*** AACGGTGTTATTTATCACAATCAG ***G*** | *Xho* I |
| S1 (491–498) F | ***GATCC*** GTGATAAATAACACCGTTGTCGGT ***C*** | *BamH* Ⅰ |
| S1 (491–498) R | ***TCGAG*** ACCGACAACGGTGTTATTTATCAC ***G*** | *Xho* I |
| S1 (493–500) F | ***GATCC*** AATAACACCGTTGTCGGTGCTATC ***C*** | *BamH* Ⅰ |
| S1 (493–500) R | ***TCGAG*** GATAGCACCGACAACGGTGTTATT ***G*** | *Xho* I |
| S1 (343–349) F | ***GATCC*** GAAACTAACTTTATGTGTACA ***C*** | *BamH* Ⅰ |
| S1 (343–349) R | ***TCGAG*** TGTACACATAAAGTTAGTTTC ***G*** | *Xho* I |
| S1 (342–348) F | ***GATCC*** CTTGAAACTAACTTTATGTGT ***C*** | *BamH* Ⅰ |
| S1 (342–348) R | ***TCGAG*** ACACATAAAGTTAGTTTCAAG ***G*** | *Xho* I |
| S1 (492–498) F | ***GATCC*** ATAAATAACACCGTTGTCGGT ***C*** | *BamH* Ⅰ |
| S1 (492–498) R | ***TCGAG*** ACCGACAACGGTGTTATTTAT ***G*** | *Xho* I |
| S1 (491–497) F | ***GATCC*** GTGATAAATAACACCGTTGTC ***C*** | *BamH* Ⅰ |
| S1 (491–497) R | ***TCGAG*** GACAACGGTGTTATTTATCAC ***G*** | *Xho* I |
| M347L+T349R F | ***GATCC*** CTTGAAACTAACTTTCTGTGCAGA ***C*** | *BamH* Ⅰ |
| M347L+T349R R | ***TCGAG*** TCTGCACAGAAAGTTAGTTTCAAG ***G*** | *Xho* I |
| V491A F | ***GATCC*** GCGATAAATAACACCGTTGTCGGT ***C*** | *BamH* Ⅰ |
| V491A R | ***TCGAG*** ACCGACAACGGTGTTATTTATCGC ***G*** | *Xho* I |
