# Supplementary material for: Identification of two immunodominant and neutralizing linear B-cell epitopes exposed on the surface of the porcine deltacoronavirus spike protein
Source: Vet Res. 2026 Jan 27;57:33. doi: 10.1186/s13567-025-01690-x (PMC12918246; doi:10.1186/s13567-025-01690-x)
Supplement: Supplementary file 3 — Additional file 3 Information on the two sets of overlapping 8-mer peptides (6-amino-acid stagger) synthesized for epitope mapping. [file 13567_2025_1690_MOESM3_ESM.docx]

**Additional file 3.** Information on the two sets of overlapping 8-mer peptides (6-amino-acid stagger) synthesized for epitope mapping.

| Peptide name | Amino-acid sequence | Location (N→C) | Application |
| --- | --- | --- | --- |
| P25590-1 | FRLETNFM | 340−347 | Mapping the epitope recognized by mAb B8F10 |
| P25590-2 | LETNFMCT | 342−349 |  |
| P25590-3 | TNFMCTGC | 344−351 |  |
| P25590-4 | FMCTGCTM | 346−353 |  |
| P25590-5 | CTGCTMNL | 348−355 |  |
| P25590-6 | TPSQLIVI | 485−492 | Mapping the epitope recognized by mAb G10C2 |
| P25590-7 | SQLIVINN | 487−494 |  |
| P25590-8 | LIVINNTV | 489−496 |  |
| P25590-9 | VINNTVVG | 491−498 |  |
| P25590-10 | NNTVVGAI | 493−500 |  |
